# Wheat Bread Enriched with House Cricket Powder (*Acheta domesticus* L.) as an Alternative Protein Source

**DOI:** 10.3390/molecules29030711

**Published:** 2024-02-03

**Authors:** Magdalena Gantner, Anna Sadowska, Anna Piotrowska, Klaudia Kulik, Barbara Sionek, Eliza Kostyra

**Affiliations:** 1 Department of Functional and Organic Food, Institute of Human Nutrition Sciences, Warsaw University of Life Sciences, Nowoursynowska Str. 159c, 02-776 Warsaw, Poland; anna_sadowska@sggw.edu.pl (A.S.); anna_piotrowska@sggw.edu.pl (A.P.); klaudia_kulik@sggw.edu.pl (K.K.); eliza_kostyra@sggw.edu.pl (E.K.); 2 Department of Food Gastronomy and Food Hygiene, Institute of Human Nutrition Sciences, Warsaw University of Life Sciences, Nowoursynowska Str. 159c, 02-776 Warsaw, Poland; barbara_sionek@sggw.edu.pl

**Keywords:** cricket powder, wheat flour, bread, sensory evaluation, fatty acids, amino acids, functional food

## Abstract

The house cricket (*Acheta domesticus* L.) is one of four edible insect species introduced to the EU market as a novel food and alternative protein source. Innovative products, such as cricket flour, are increasingly appearing on supermarket shelves and can offer an alternative to traditional cereals, while providing the body with many valuable nutrients of comparable quality to those found in meat and fish. The aim of this study was to investigate the possibility of using cricket powder as a substitute for wheat flour in the production of bread. The physicochemical properties of cricket powder were evaluated in comparison to wheat flour. As a result of technological studies, bread compositions with 5%, 10% and 15% replacements of wheat flour by cricket powder were designed and their quality characteristics (physicochemical, sensory and microbiological) were evaluated. Cricket powder was characterised by a higher protein (63% vs. 13.5%) and fat (16.3% vs. 1.16%) content and a lower carbohydrate (9.8% vs. 66%) and fibre (7.8% vs. 9.5%) content as compared to wheat flour. The tested preparations had a similar pH (6.9 and 6.8, respectively, for cricket powder and flour) and fat absorption capacity (0.14 vs. 0.27 g oil/g powder, respectively, for cricket powder and flour) but different water holding capacities and completely different colour parameters. All breads had good microbiological quality after baking and during 7 days of storage. In instrumental tests, the 10 and 15% replacements of wheat flour by cricket powder affected the darker colour of the breads and caused a significant increase in the hardness of the breads. The research has shown that the optimal level of replacement, which does not significantly affect the physiochemical and sensory characteristics, is 5% cricket powder in the bread recipe. Considering the results obtained and the fact that insects provide a sufficient supply of energy and protein in the human diet, are a source of fibre, vitamins and micronutrients, and have a high content of monounsaturated and polyunsaturated fatty acids, the suitability of cricket powder for protein enrichment of bakery products is confirmed.

## 1. Introduction

The growing world population, depletion of freshwater resources, climate change, environmental pollution, and increasing costs of livestock production are the most common factors contributing to the increased search for alternative food sources that are safe for humans and cheap to produce [1]. Edible insects are a rich source of nutrients, especially protein. They also have a high feed conversion efficiency compared to conventional livestock. Many species can be raised on organic waste, increasing their sustainability. Insect farming has a much lower environmental impact than conventional livestock farming. They emit fewer greenhouse gases and use less water and land. This is particularly important in the context of climate change and increasing pressure on natural resources. Edible insects can be farmed intensively on a relatively small area, which is particularly beneficial in urban or arid environments where traditional agriculture is challenging. In some of the world’s most vulnerable regions, insect farming can provide a sustainable food source that is critical to food security. Insects therefore have the potential to become a more important part of the human diet in the future. It should be noted, however, that in many parts of the world where eating insects is not currently the norm, there are cultural and regulatory hurdles to overcome [2,3]. Insect consumption—entomophagy—is an integral part of the diet of at least 2 billion people in Asia, Latin America, Africa and Australia [4,5]. More than 2000 species of edible insects are consumed worldwide [6]. The most commonly consumed species are those belonging to the orders Coleoptera (31.2%), Lepidoptera (17.1%), Hymenoptera (bees, wasps, ants, 15.2%), Orthoptera (grasshoppers, crickets, locusts, 13.2%) and Hemiptera (11.2%) [7]. Contrary to popular belief, insects are not only consumed in times of food scarcity. In many cultural circles (local culinary traditions), they are eaten by choice, both for their taste and their high nutritional value [7,8]. Insects provide high-quality protein and nutrients comparable to meat and fish. The protein and fat content of insects varies from 25 to 75 percent and 10 to 70 percent in dry matter, respectively, and depends on the species and life stage, as well as environmental factors such as feeding, temperature and light [9]. Most insect species are rich in fatty acids. They are also rich in fibre and micronutrients such as copper, iron, magnesium, manganese, phosphorus, selenium and zinc, as well as vitamins such as riboflavin, pantothenic acid, biotin and, in some cases, folic acid and carotenoids [7,8,9,10,11,12]. Considering both their nutritional properties, such as their high protein digestibility, breeding considerations and relatively low environmental impact, and the possibility of intensive production in places where traditional agricultural production is not possible, insects appear to be the future of human nutrition [13,14,15,16]. 

As a result, the European Commission has taken steps to standardise edible insects from 2021. Currently, the insect species authorised as novel foods in the EU are *Tenebrio molitor* L., *Locusta migratoria* L., *Acheta domesticus* L. and *Alphitobius diaperinus* Panzer [17,18,19,20,21]. A further seven species are awaiting review. One of the edible insects that are farmed is *A. domesticus* [22]. Its protein and lipid contents are 20–25 and 4–7 g/100 g fresh weight, respectively, which is comparable to the content of these components in beef or chicken [23]. Its fat composition is 29–31% polyunsaturated fatty acids, and this insect is also a valuable source of vitamins [2]. The production efficiency of *A. domesticus* is 2.1, meaning that 2.1 kg of dry feed is required to produce 1 kg of food. In comparison, it takes 4.5 to produce 1 kg of edible product from poultry, 9.1 from pork and 25 kg of feed from beef [1]. House cricket is used for both food and feed purposes [24]. House cricket powder obtained from dried insects contains about 56% protein and about 27% fat with a predominance of C16:0 and C18:2 (*cis*-9,12) fatty acid fractions [23]. Food producers in the EU can now use whole individuals of A. domesticus in frozen, dried, powdered and partially defatted form in the production of many food products, including cereal bars, breads and rolls, crackers, dry-pasta-based products, meat analogues, soups and soup concentrates, confectionery or processed meat products. 

Currently, the main problem limiting the use of insects in the human diet in most European countries is the lack of acceptance and safety of this type of food. The main obstacle is undoubtedly cultural barriers and the associated lack of acceptance, reluctance or even fear among potential consumers. In contrast to the attitudes of populations in cultures where various insect species are considered traditional delicacies, consumers in European countries react with revulsion at the prospect of consuming organisms that are not commonly known as food, but as pests [25]. However, the introduction of insects into the diet does not necessarily mean consuming them in their natural form. There are many ways to process insects into a form that is more socially acceptable in developed countries, such as flour (powder), paste or extract (protein isolate). Studies by Tan et al. [25] and Hartmann and Siegrist [26] show that the way such products are prepared has a strong influence on their sensory acceptance by consumers who do not consume insects on a daily basis. According to de Oliveira et al. [27], a form reminiscent of conventional food, such as an accompaniment to minced meat or bread, was most accepted by consumers compared to eating insects whole.

According to the data cited above, cricket powder has a high nutritional value. However, its use in food production as a substitute for bread flours poses technological challenges due to the lack of gluten, as well as the possibility of specific sensory characteristics (such as a bitter aftertaste). Therefore, the purpose of the present study was to investigate the possibility of developing a bread formulation with the replacement of wheat flour with cricket powder at three levels, i.e., 5%, 10% and 15%, while maintaining an optimal nutritional value, level of physicochemical properties and overall sensory quality.

## 2. Results and Discussion

### 2.1. Characteristics of Cricket Powder and Whole Wheat Flour

The chemical composition of whole wheat flour and cricket protein powder used for bread making is presented in Table 1. The protein content in cricket powder was about 63 g/100 g, several times higher than in wheat flour (13.5 g/100 g). Cricket powder was also characterised by a higher fat content as compared with wheat flour (16.29 vs. 1.16 g/100 g). However, wheat flour had a higher fibre content (9.50 vs. 7.80 g/100 g) and a much higher carbohydrates content (66.04 vs. 9.83 g/100 g) than cricket protein. Similar results on the chemical composition of cricket powder were presented by Rumpold and Schüter, Lucas-González et al. and Mafu et al. [2,28,29]. As confirmed in a study by Kowalski et al. (2022) [30], edible insects are considered to be a food source with high nutritional value due to their high content of proteins, fats, vitamins and minerals. 

**Figure 1:**
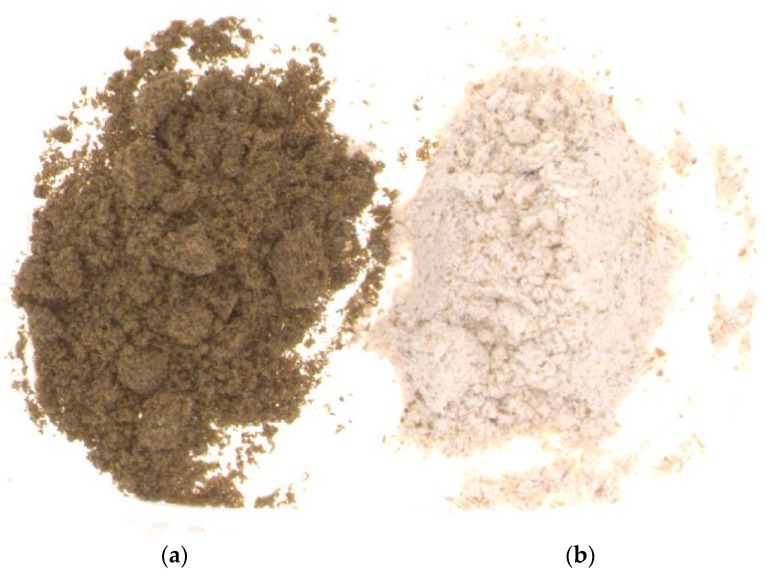
Pictures of cricket protein powder (**a**) and wholemeal wheat flour (**b**) used for bread making.

The tested flours differed statistically significantly (*p* < 0.05) in terms of water holding capacity, water activity and colour parameters, while they were similar in terms of pH and fat absorption (Table 2). Wheat flour had lower water absorption properties of about 1.52 g water/g flour compared to a mean value of about 2.53 g water/g obtained for cricket powder. The differences obtained in water absorption properties may be due to the higher protein content of cricket powder 63 g/100 g compared to wheat flour—13.5 g/100 g. The water activity parameter ranged from 0.18 for cricket powder to 0.43 for wheat flour. According to Tiwari and Jha [31], a low a_w_ value in the range of 0.1 to 0.33 is favourable for the microbiological stability of products, which facilitates storage and distribution. The L* colour parameter values of cricket powder and wheat flour were 35.14 and 81.58, respectively. Wheat flour had a much lighter colour, which was easily visible to the naked eye (Figure 1). The other colour parameters, a* and b*, also showed significant variations (*p* < 0.05). The colour parameter a* values obtained for cricket powder and wheat flour were 4.56 and 4.74, respectively, and the parameter b* values for cricket powder and wheat flour were 25.22 and 21.58, respectively. The results show that wheat flour was characterised by a slight colour shift towards a red hue compared to cricket powder, which in turn showed a greater colour shift towards a yellow hue. In addition, as can be seen in Figure 1, wheat flour was characterised by a high particle size in contrast to cricket powder, where chitin elements were very prominent. 

A similar result was reported by other researchers, including Mafu et al. [29], who stated that cricket powder showed a low value of lightness L* (40.81 ± 0.61) and redness a* (4.9 ± 0.53) and a high value of yellowness b* (15.13 ± 2.02). In another study, Lucas-González et al. [28] reported that cricket powder showed a low value of L* (41.62 ± 0.61), caused by the brown colour of the outer chitin skeleton, which is characteristic of insects.

The protein powder obtained from cricket was characterised by a higher content of saturated fatty acids (about 35% of fat content) and monounsaturated fatty acids (about 26% of fat content), and a much lower level of polyunsaturated fatty acids (about 34% of fat content) in comparison with the wheat flour sample (the content of the above-mentioned fatty acid groups was about 16%, 14%, 64% of fat content, respectively) (Table 3). In addition, wholemeal wheat flour showed a more than threefold higher content of fatty acids from the n-3 family (about 3.6% of fat content vs. 1.0% of fat content) and almost twice the content of acids from the n-6 family (about 60.5% of fat content vs. 32.1% of fat content). The fatty acid profile of cricket powder is similar to that of pork [32].

Due to the approximately five times higher protein content in the cricket powder (about 63%) compared to the content of this macronutrient in the wholemeal wheat flour (about 13%), the content of all amino acids tested was also higher in the cricket powder. The amino acid content of the cricket powder was between two (for glutamic acid) and twelve (for alanine) times higher than their content in the wheat flour (Table 4).

### 2.2. Assessment of Quality Parameters of Designed Cricket Bread

#### 2.2.1. Physicochemical Properties

Table 5 shows the physical parameters, i.e., the colour components, specific gravity, water activity and hardness of the breads with 5, 10 and 15 percent additions of cricket powder and the control bread. Statistically significant (*p* < 0.05) differences were obtained for most of the parameters tested, except density and water activity.

Colour is one of the most important characteristics of bakery products and, along with texture, can influence consumer acceptance. The values of the colour component L* ranged from about 40 for bread with 10 and 15% cricket powder additions to about 44–45 for the control sample and with 5% cricket powder addition. The results obtained indicate that the addition of cricket powder significantly (*p* < 0.05) affects the darker colour of the bread crumb; however, the lowest level of addition (5%) does not cause significant changes in the brightness of the bread. The values of the colour parameters a* and b* were significant (*p* < 0.05) and highest in the control sample, meaning it had the most intense red and yellow colour compared to the samples with added cricket powder. Similar results were obtained in studies by Gantner et al. [33] and Khuenpet et al. [34] on the effect of adding mealworm powder on the colour of bread and muffins. In these authors’ study, the addition of mealworm powder contributed to a significant (*p* < 0.05) reduction in the red and yellow colour of the tested bakery products compared to the control. These results are also in line with the study of Bartkiene et al. [35], where the addition of cricket flour reduced dough redness and yellowing. The colour changes in a* and b* of the bread crumb may be due to the natural colour of the whole wheat flour, while the differences in the L* parameter are due to the darker brown colour of the cricket chitin. González et al. [36] reported that the colour of bakery products directly depends on the colour of raw material.

In this study, the total colour difference (ΔE) and the browning index (BI) were calculated from the L*a*b* colour parameters studied. The results obtained show an increase in the value of ΔE as the proportion of insect powder in the bread composition increased. Based on the study of Bellary et al. [37], it is known that the colour difference ΔE ˃ 3.0 is visually noticeable to inexperienced consumers. In the present study, the values of the total bread colour difference (ΔE) ranged from 1.03 for the bread with the lowest addition of cricket powder to 6.27 for the sample with 10% addition. These results indicate that the breads developed with 10 and 15% cricket powder additions have significant colour differences compared to the control sample, which may result in lower consumer acceptability compared to traditional wheat breads. No effect of cricket powder addition on the BI value was observed, which may mean that the contribution of the component with a higher protein content (cricket powder vs. bread flour) did not contribute to the intensity of the Maillard reaction that occurs during the baking process.

The hardness parameter showed that the textural values of the bread were significantly influenced by the addition of 10 and 15% cricket powder. The differences obtained may have been due to the different content of individual nutrients (fat, fibre, gluten protein) in the samples tested, which shaped the texture of the bread in different ways. The lowest addition of 5% insect powder did not significantly (*p* > 0.05) affect hardness compared to the control bread. These results partially confirmed the study by Kowalski et al. [30], where a decrease in bread hardness was observed at a 10% addition of cricket flour, with a significant increase in the parameter studied at 20 and 30%. Similar results were presented by Khuenpet et al. [34]. As reported by Villarino et al. [38], bread production is sensitive to replacing wheat flour with gluten-free, non-starchy flour, particularly by disrupting the development of gluten. According to de Oliveira et al. [27] and González et al. [36], a high hardness of the bread indicated the resistance to deformation and long time needed to chew the bread product before swallowing. On the other hand, García-Segovia et al. [39] and Gonzalez et al. [36] reported no significant differences in the hardness of breads where wheat flour was replaced by insect flour. No significant differences in terms of water activity and density were observed among the analysed samples, 0.95–0.96 and 0.39–0.45, respectively. It is likely that the composition of the flour, concerning proteins and carbohydrates, may be responsible for interfering with the proper development of the dough during fermentation, causing a poor gas-holding capacity of the resulting weak gluten networks, giving a compact bread crumb structure [40]. 

No significant differences in terms of water activity were observed among the analysed samples, being 0.95–0.96. Different results were obtained by Ruszkowska et al. [41], where snacks with 2 and 4% cricket powder added had a lower water activity compared to the control sample, which directly affected their crispness. In a study by Mafu et al. [29], bread with 10% and 25% cricket powder had the highest moisture content and was significantly different (*p* < 0.05) from the control bread. According to the authors, who did not show a linear effect of insect protein addition on bread moisture content, the dough mixed with gluten protein and cricket protein particles is gradually but inconsistently moistened compared to that without cricket powder. This may account for the variability in moisture content of the fortified bread, which should be investigated in the future, especially since this parameter is closely related to the hardening process of starch products [39].

#### 2.2.2. Nutritional Value

Replacing part of the flour with a cricket protein preparation resulted in obtaining breads with higher protein and fat contents as compared to the control sample (Table 6). Breads with 10 and 15% cricket protein additions showed more than 20% of energy value obtained from protein, thus meeting the requirements for products with high protein content, according to Regulation EU No. 1924/2006 of the European Parliament and of the Council of 20 December 2006 on nutrition and health claims [42]. A higher protein content also results in higher levels of amino acids, which was confirmed in the studies of Mafu et al. [29] and Wieczorek et al. [43], who observed a significant increase in the content of essential amino acids after baking breads with cricket powder addition. With an increase in the addition of cricket protein preparation to the breads, a decrease in carbohydrate content and a slight decrease in dietary fibre were observed compared to the control bread. Nevertheless, the decrease in fibre content was not so significant, so the developed breads could not be labelled with the following nutrition claim: source of fibre is in accordance with the regulation stated above [42].

House crickets (*Acheta domesticus* L.) and other edible insects have gained attention not only for their high nutritional value, but also for showing health benefits due to their content of bioactive compounds. Some studies [44,45] suggest that edible insects, including crickets, may have antioxidant properties. Antioxidants help neutralise harmful free radicals in the body, potentially reducing oxidative stress and inflammation. Also, the insect exoskeleton is composed of chitin, a fibrous substance. Chitin may have potential health benefits, such as supporting gut health and modulating the immune system. Moreover, insects are highly sustainable and environmentally friendly sources of protein. They require fewer resources (water, feed and space) compared to traditional livestock, contributing to sustainable food production. Chitin and other components of insect exoskeletons can act as prebiotics, promoting the growth of beneficial gut bacteria [46].

#### 2.2.3. Sensory Evaluation

**Figure 2:**
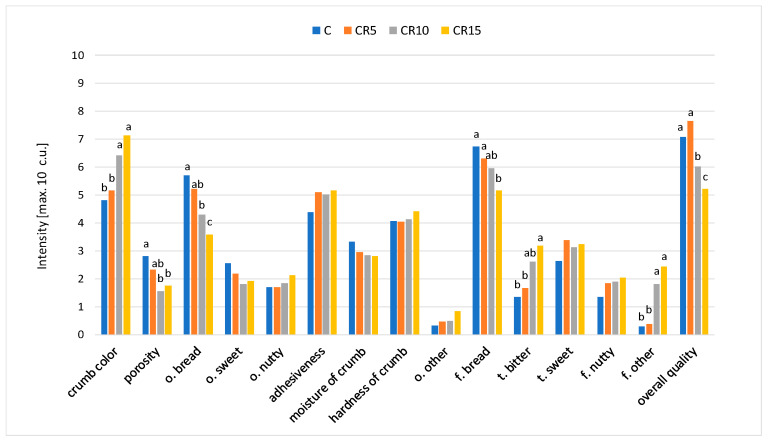
The sensory characteristic of the breads with the addition of cricket powder (f.—flavour; t.—taste; o.—odour). Letters a–c indicate significant differences in intensity of attributes between the evaluated samples (*p* < 0.05).

The results of sensory evaluation revealed that the addition of cricket powder influenced the visually assessed colour and porosity of the samples (Figure 2). The intensity of bread crumb colour increased and the porosity decreased with a higher cricket powder addition. The crumb of CR10 and CR15 samples was significantly darker and less porous than the control sample. Colour is an important bread feature influencing consumer choice and acceptance. A significant effect of insect powder addition on the colour of bread and other bakery products was also demonstrated in studies by other authors [30,33,36]. The influence on crumb porosity may be due to the fact that cricket powder does not contain gluten [47]. Moreover, cricket powder is made from adult insects, having an exoskeleton, head, eyes, lower jaw, antennae, legs and wings. As a consequence, it contains some amounts of chitin and is also characterised by a coarse-grained structure, which may affect the baking properties of the flour [48].

Visually perceived differences in the porosity of the crumb did not affect the consistency attributes assessed in the oral cavity. All bread samples were described as quite soft, moderately adhesive, and moist, regardless of the cricket powder addition level. These results are in line with González et al.’s [36] studies which showed that replacing wheat flour with cricket powder at the level of 5% did not significantly affect the bread texture parameters measured instrumentally. Nevertheless, replacing wheat flour with cricket powder at levels of 10 and 15% contributed to an increase in crumb hardness in instrumental evaluation, but this did not result in an increase in oral feeling as assessed by experts in sensory tests. 

With regard to odour attributes, bread enrichment with the cricket powder influenced the perceptibility of the bread odour, the intensity of which was significantly higher in the control sample compared to CR10 and CR15. The intensity of the sweet and nutty odour remained at a similar level, independent of the addition of cricket powder. Recipe modification also influenced the intensity of the bread flavour and bitter taste. The CR15 sample, compared to the control sample, was characterised by a significantly lower intensity of the bread flavour and a higher intensity of the bitter taste. The intensity of the sweet and nutty flavours was similar in all bread samples. In breads with a higher level of cricket powder addition (CR10 and CR15), the presence of an off-flavour unusual for bread was found. As indicated in the literature, cricket powder is characterised by a strong flavour described as crustacean-like, cooked-legumes-like and earthy [48]. Therefore, too high a level of cricket powder addition to bread negatively affects the sensory quality.

The differences in the intensity of sensory attributes affected the assessment of the overall bread sensory quality, defined as an appropriate harmonisation of sensory features. The highest sensory quality, not significantly different from the control sample, was characterised by the CR5 sample. The sensory quality of the CR10 and CR15 samples was statistically significantly lower than the control sample. Enriching bakery products with powder forms of edible insects is challenging, as fortification impacts the sensory properties of the final product with a consequent reduction in palatability and overall consumer acceptability. Thus, including insect flours without negatively altering sensory quality is only possible to a certain level. Osimani et al. [49] found that the addition of cricket powder at the levels of 10%, 20% and 30% markedly affected the acceptance of the bread due to the notable flavour and taste of the insect-based ingredient. The highest degree of palatability was found for the control bread, while the samples of bread with the addition of cricket powder at the levels of 20 and 30% were not accepted by consumers.

#### 2.2.4. Microbiological Evaluation

The results of microbiological analysis of the tested breads during 7 days of storage are presented in Table 7. Total viable counts (TVCs) after baking in all of the examined breads were low (from 3.71 log CFU g^−1^ for control sample to 4.52 log CFU g^−1^ for CR10 sample). There were no statistically significant differences (*p* > 0.05) in TVC between the breads with different amounts of insect powder. After 2 days after baking, there was an increase in TVC in all variants of the breads of about 2–3 logarithmic orders. A high level of Total viable counts (from 7.37 log CFU g^−1^ for CR5 to 9.44 log CFU g^−1^ for CR15 sample, respectively) was found in the control, as well as in breads with insect powder at 7 days of storage. Malomo et al. [50] also observed a similar increase in Total viable counts to approx. 8–9 CFU g^−1^ in the breads with 0, 1 and 3% of cottage cheese (warancashi), stored for 9 days under ambient temperature. 

Yeasts and moulds are the main microorganisms responsible for the deterioration of bread [51]. The water activity of bread (0.95–0.96), moisture content (about 40%), pH (5–6) and presence of nutrients favour fungal development [52,53,54,55,56,57,58,59]. Fungal spoilage affects the safety aspects due to mycotoxigenic fungi and the limited shelf life of bread, contributes to economic losses and household bakery products waste, and decreases sensory quality [52,60]. 

There was no presence of yeasts and moulds after baking in the breads with and without the addition of insect powder. The high temperature during the bread-making process eliminates natural microbiota from the raw material [61]. Bread recontamination with yeasts and moulds is possible after baking at the packing, cooling and storage stage [62,63]. Special attention must be paid to the hygienic quality of the air due to the fungal particles dispersed [63]. After 7 days of storage, all tested breads were characterised by non-statistically significant and relatively low counts of yeasts and moulds (approx. 4 log CFU g^−1^).

## 3. Materials and Methods

### 3.1. Materials

The tested materials were cricket powder, wholemeal wheat flour and breads, where wholemeal wheat flour was replaced with cricket powder at levels of 5, 10 and 15%. For the preparation of wheat bread (control sample) and wheat bread with insect flour, the following materials were used: commercial whole wheat bread flour type 1850 (Melvit, Warszawa, Poland), salt, dried yeast purchased on the local market and cricket (*A. domesticus*) protein powder produced by SENS Food LTD (London, UK). 

### 3.2. Methods

#### 3.2.1. Functional Properties of Cricket Powder and Whole Wheat Flour

##### pH Measurement

The pH was measured using the potentiometric method with a portable pH meter (model 205, Testo AG, Lenzkirch, Germany). The pH values were measured in a 5% solution of wheat flour and cricket powder in water. The tests were measured in triplicate.

##### Water Activity (a_w_) Measurement

To measure a_w_, 5 g of whole wheat flour and cricket powder were placed in a measuring cup in the device (AquaLab 4TEV, Munich, Germany). The tests were measured in triplicate.

##### Water Holding Capacity (WHC) Measurement

WHC analysis was performed on 1 g of cricket protein powder or whole wheat flour. The sample was homogenised using an ultrasonic homogeniser (Hielscher UP400ST, Berlin, Germany) with 30 cm3 of distilled water and then centrifuged for 15 min (6000 RPM, 0 °C) in a centrifuge (MPW-380 R, MPW Med. Instruments, Warsaw, Poland). After flooding with complementary water, samples with sediment were left upside-down for 10 min and then weighed. The WHC was determined in triplicate for each sample by weighing the samples at the beginning and after centrifugation by calculating the difference in mass. The measurement was expressed as grams of water absorbed per gram of flour or powder.

##### Fat Absorption Capacity (FAC) Measurement

FAC was determined using a similar procedure but with rapeseed oil. The FAC was expressed as the amount of oil per gram of flour or powder and was determined in triplicate.

##### The Colour Measurement

The IRIS artificial eye visual analyser (AlphaMos, Tuluse, France) was used to measure wholemeal wheat flour, cricket powder and bread colour. Colour measurements were carried out triplicate. The loaf was randomly selected from the batch. The colour components were read in the same way as for the flour measurements. Colour was recorded on the CIE L*a*b* scale in terms of brightness (L*) and colour (a*—redness; *—yellowness). In addition, the total colour difference (ΔE) between the reference sample and the test sample was calculated using the following equation: ΔE = √ΔL^2^ + Δa^2^ + Δb*^2^. The browning index was also calculated using following equation:

$$\mathrm{BI}=\underset{o}{\frac{100\;\left(x-0.31\right)}{0.15}}\;\mathrm{BI}=\underset{o}{\frac{100\;\left(x-0.31\right)}{0.15}}\;\mathrm{where}\;x=\underset{l}{\frac{a^{*}+1.75L}{5.645L^{*}+a^{*}-0.12b^{*}}}$$

##### Chemical Composition

Chemical tests on cricket powder and wholemeal wheat flour assessed water, protein, fat, dietary fibre, amino acid and fatty acid profiles. These tests were carried out in an accredited analytical laboratory according to the test procedures used there:

- The water content was determined using the gravimetric method;
- The fat content was determined using the Soxhlet method (extraction-weighing method);
- The amino acids profile was determined using LC-MS/MS;
- The protein content was calculated based on the analysis of the content of individual amino acids;
- The fibre content was determined by the enzymatic-weighing method;
- The fatty acids profile was determined using gas chromatography with flame ionisation detection (GC-FID).

#### 3.2.2. Development of the Composition of Bread with the Addition of Cricket Preparation

The bread was made in three variants with different amounts of cricket protein powder, added to partially replace whole wheat flour at 5, 10 and 15%. The control bread was made without the addition of insect powder. Water, dried yeast, salt and sugar were placed in a mixing bowl and mixed for 3 min at 37 °C. Then, flour was added, and the dough was mixed for 3 min. The dough was then left to rise in a warm place for 1 h. Table 8 shows the complex combination of the samples tested.

#### 3.2.3. Physical Parameters of Designed Breads

##### Density Measurement of Prepared Breads

The density of bread was determined by pouring it into a measuring cup filled with seeds. A slice of bread was placed in the centre. By observing the increase in the volume of the mixture and knowing the mass of the sample, the density of the samples tested was determined. The measurement was carried out in three repetitions, for each bread sample. The density of bread was calculated using the following equation:

$$\mathrm{Density}\;\underset{o}{\left[\frac{g}{{\mathrm{cm}}^{3}}\right]}=\frac{\mathrm{loaf}\;\mathrm{weight}\;\left[g\right]}{loaf\;volume\;\left[{\mathrm{cm}}^{3}\right]}$$

##### Hardness Measurement of Prepared Breads

The mechanical properties of the crumb, expressed as hardness, were measured on the discs with a thickness of 20 m using a texture analyser (Stable Micro Systems TA. XT2i, Hamilton, MA, USA). The settings used were as follows: 25 mm diameter cylindrical aluminium probe, 50% deformation, 5 s pause between measurements and 5 mm/s probe movement speed. Measurements were made in six replicates at room temperature. Three slices of each loaf (from the centre) were measured. 

#### 3.2.4. Nutritional Value Assessment of Prepared Breads

The nutritional value, i.e., protein, fat, fibre and carbohydrate content, of the designed breads was calculated on the basis of the chemical composition of the components both after analytical determinations (cricket powder and wheat flour) and on the basis of the nutritional value tables (other components) [64].

#### 3.2.5. The Microbiological Evaluation of Prepared Breads

The evaluation was performed using the plate method. Total viable counts (TVCs) were determined according to ISO 4833 1:2013-12/A1:2022-06 [65] on nutrient agar (Merk, Germany). Plates were incubated at 30 °C for 72 h. The total number of yeasts and moulds (Y&M) was analysed according to ISO 21527-1:2008 [66] on YGC agar (Sabouraud Dextrose with Chloramphenicol LAB-Agar, Biomaxima, Lublin, Poland). Plates were incubated at 25 °C for 72–120 h. The results of the viable counts are expressed as mean of log CFU g^−1^ ± standard deviation. The tests were measured in triplicate.

#### 3.2.6. Sensory Evaluation

For the sensory assessment of bread samples, the scaling method according to ISO 4121:2003 [67] was used. The evaluation was performed by a trained panel (n = 6) fulfilling the requirements of ISO standard 8586:2012 [68]. The assessors evaluated the intensity of attributes in two sessions using a 10 cm linear unstructured scale ranging from “none” to “very strong” (for odour, flavour and taste descriptors) and “low” to “high” for texture attributes evaluated in the oral cavity (adhesiveness, moisture of crumb and hardness of crumb), as well as the overall quality of the samples. For visual attributes, crumb colour was assessed on a scale ranging from “light” to “dark”, whereas crumb porosity was evaluated on a scale anchored from “low porosity” to “high porosity”. The sensory overall quality was defined as the impression of the harmony of all evaluated attributes, with either no or only a slight intensity of negative descriptors. Since the evaluation of bread samples was performed in two independent sessions (repetitions), the average values of each sensory attribute were based on twelve individual results. Individual bread samples of the same size were presented in transparent plastic containers (200 mL) coded with 3-digit numbers and covered with lids. Still mineral water was used as a taste neutraliser. The order of sample presentation was balanced to account for first-order and carry-over effects. The assessment was performed in the sensory laboratory equipped with individual booths with white light. The laboratory met general requirements of ISO standard 8589:2010 [69].

#### 3.2.7. Statistical Analysis

Statistica 13.0 software (Tibco Software Inc., Palo Alto, CA, USA) was used to statistically process the data obtained from the instrumental tests. To determine whether there were statistically significant differences in the physicochemical parameters of the cricket preparation and wheat flour, the t-Student test was applied at the level of significance of *p* < 0.05. To find statistically significant differences between the studied parameters of the obtained breads, analysis of variance (ANOVA) was used for dependent groups with post hoc analysis of Duncan’s test at a significance level of *p* < 0.05. Any differences between the groups at the significance level of *p* < 0.05 were considered statistically significant.

XLSTAT version 2021 (Addinsoft Software, Paris, France) was used for sensory data statistical analysis and interpretation. Two-way ANOVA with Fisher’s Least Significant Difference (LSD) test with post hoc analysis (*p* < 0.05) was used to find the differences between the bread samples in the intensity of the examined attributes.

## 4. Conclusions

The results of the present study indicate that cricket protein powder can be used for the production and protein enrichment of wheat bread. The cricket powder addition variants used in all cases increased the protein and fat content and decreased the carbohydrate content of the product. The fortification of wheat bread with cricket flour at 10 and 15 percent affected the colour components and caused a significant increase in hardness measured instrumentally, which did not lead to an increase mouthfeel as assessed by sensory evaluation. For most of the physicochemical parameters and sensory evaluation, the breads with 5% cricket flour did not show any significant differences compared to the control sample. All breads had a good microbiological quality after baking and during 7 days of storage. Considering the results obtained and the fact that insects provide a sufficient supply of energy and protein in the human diet, are a source of fibre, vitamins and micronutrients, and have a high content of monounsaturated and polyunsaturated fatty acids, the suitability of cricket powder for the protein enrichment of bakery products is confirmed. Future research should focus on the potential safety problems of edible insects (allergic reactions and contamination with pathogenic micro-organisms) and the development of appropriate recipes, in order to create a positive consumer attitude towards innovative insect-based foods.

## Figures and Tables

**Table 1:** Chemical composition of cricket protein powder and whole wheat flour.

Nutrients Content	Cricket Protein Powder	Whole Wheat Flour
Fat [g/100 g]	16.29 ± 3.10	1.16 ± 0.23
Protein [g/100 g]	63.00 ± 7.56	13.5 ± 1.62
Carbohydrates [g/100 g]	9.83 ± 0.95	66.04 ± 1.43
Fibre [g/100 g]	7.80 ± 1.20	9.5 ± 1.5

**Table 2:** Functional properties of cricket protein powder and whole wheat flour.

Physicochemical Properties	Cricket Powder	Whole Wheat Flour
pH	6.90 ± 0.02	6.77 ± 0.13
Fat absorption capacity [g oil/g powder]	0.14 ± 0.12	0.27 ± 0.05
Water holding capacity [g water/g powder]	2.52 ± 0.54 ^b^	1.52 ± 0.14 ^a^
a_w_	0.18 ± 0.00 ^a^	0.43 ± 0.00 ^b^
L*	35.14 ± 0.53 ^a^	81.58 ± 1.34 ^b^
a*	4.56 ± 0.10 ^a^	4.74 ± 0.48 ^b^
b*	25.22 ± 0.09 ^b^	21.57 ± 0.98 ^a^

Values followed by different small letters (^a^,^b^) in the same row are significantly different (*p* < 0.05). Values are means of three replicates ± standard deviation.

**Table 3:** Fatty acid profile of cricket protein powder and whole wheat flour.

Fatty Acid Profile	Cricket Protein Powder	Whole Wheat Flour
Saturated fatty acids [g/100 g of fat content]		
(C14:0) myristic acid	0.50 ± 0.20 *	0.06 ± 0.03
(C15:0) pentadecanoic acid	0.08 ± 0.04	0.06 ± 0.03
(C16:0) palmitic acid	23.98 ± 4.80	14.80 ± 2.96
(C17:0) margaric acid	0.15 ± 0.06	0.07 ± 0.03
(C18:0) stearic acid	9.66 ± 1.94	0.97 ± 0.30
(C20:0) arachidic acid	0.29 ± 0.12	0.12 ± 0.05
(C22:0) behenic acid	<0.05	0.17 ± 0.07
(C24:0) lignoceric acid	<0.05	0.14 ± 0.06
Monounsaturated fatty acids [g/100 g of fat content]		
(C16:1w7) palmitoleic acid	0.67 ± 0.21	0.12 ± 0.05
(C18:1w9) oleic acid	24.64 ± 4.93	12.99 ± 2.60
(C18:1w7) *cis*-11-vaccenic acid	0.60 ± 0.18	1.03 ± 0.21
(C18:1w9t) *trans* elaidic acid	0.12 ± 0.05	<0.05
Polyunsaturated fatty acids [g/100 g of fat content]		
(C18:2w6) linoleic acid (LA)	32.10 ± 6.42	60.53 ± 12.11
(C18:2 ct) *cis*-9, *trans*-12 octadecadienoic acid	0.58 ± 0.18	<0.05
(C18:2w6t) *trans* linolelaidic acid	0.13 ± 0.06	<0.05
(C18:2 tc) *trans*-9, *cis*-12 octadecadienoic acid	0.68 ± 0.21	<0.05
(C18:3w3) *cis*-9, 12,15 alpha-linolenic acid (ALA)	0.99 ± 0.30	3.64 ± 0.73
(C20:2) *cis*-11,14-eicosadienoic acid	0.43 ± 0.18	0.08 ± 0.04
Contribution of individual fatty acid groups [g/100 g of fat content]		
Saturated fatty acids	34.66 ± 6.94	16.39 ± 3.28
Monounsaturated fatty acids	25.91 ± 5.19	14.30 ± 2.86
Polyunsaturated fatty acids	33.52 ± 6.71	64.25 ± 12.85
Trans fatty acids	1.51 ± 0.31	<0.55 ± 0.17
Omega 3 fatty acids (ALA, EPA, DHA, ETE, DPA) **	0.99 ± 0.30	3.64 ± 0.73
Omega 6 fatty acids (LA, GLA, ARA, DGLA) ***	32.10 ± 6.42	60.53 ± 12.11

(*) result obtained ± extended measurement uncertainty (relative) with *p* < 0.05; (**) EPA—(C20:5w3) *cis*-5,8,11,14,17-eicosapentaenoic acid; DHA—(C22:6w3) *cis*-4,7,10,13,16,19-docosahexaenoic acid; ETE—(C20:3w3) *cis*-11,14,17-eicosatrienoic acid; DPA—(C22:5w3) cis-7,10,13,16,19-docosapentaenoic acid; (***) GLA—(C18:3w6) gamma-linolenic acid; ARA—(C20:4w6) arachidonic acid; DGLA—(C20:3w6) *cis*-8,11,14-eicosatrienoic acid.

**Table 4:** Amino acid profile of cricket protein powder and whole wheat flour.

Amino acid Profile	Cricket Protein Powder	Whole Wheat Flour
Aspartic acid	5.31 ± 0.02 *	0.64 ± 0.02
Threonine	2.37 ± 0.02	0.36 ± 0.02
Serine	2.69 ± 0.02	0.63 ± 0.02
Glutamic acid	6.98 ± 0.02	3.91 ± 0.02
Proline	3.66 ± 0.02	1.31 ± 0.02
Glycine	3.01 ± 0.02	0.51 ± 0.02
Alanine	5.32 ± 0.02	0.45 ± 0.02
Valine	3.57 ± 0.02	0.54 ± 0.02
Methionine **	1.08 ± 0.02	0.19 ± 0.02
Isoleucine	2.43 ± 0.02	0.43 ± 0.02
Leucine	4.69 ± 0.02	0.89 ± 0.02
Tyrosine	3.66 ± 0.02	0.37 ± 0.02
Phenylalanine	2.38 ± 0.02	0.61 ± 0.02
Lysine	3.57 ± 0.02	0.34 ± 0.02
Histidine	1.41 ± 0.02	0.30 ± 0.02
Arginine	3.90 ± 0.02	0.63 ± 0.02
Taurine	0.22 ± 0.02	<0.02
Hydroxyproline	0.04 ± 0.02	<0.02
Cyst(e)ine, calc. from cysteic acid	0.65 ± 0.02	0.29 ± 0.02

(*) result obtained ± extended measurement uncertainty (relative) with *p* = 95%; k = 2, k-expansion factor; (**) calc. from methionine sulfone.

**Table 5:** Physicochemical properties of obtained bread loaves.

Physicochemical Properties	C	CR5	CR10	CR15
L*	45.07 ± 0.22 ^b^	44.24 ± 0.68 ^b^	40.42 ± 0.68 ^a^	40.65 ± 0.56 ^a^
a*	6.20 ± 0.07 ^c^	5.78 ± 0.12 ^b^	5.11 ± 0.06 ^a^	5.88 ± 0.12 ^b^
b*	27.89 ± 0.01 ^c^	27.11 ± 0.09 ^b^	25.11 ± 0.52 ^a^	26.78 ± 0.10 ^b^
ΔE	-	1.03	6.27	4.15
Density [g/cm^3^]	0.44 ± 0.03	0.45 ± 0.03	0.39 ± 0.06	0.42 ± 0.08
a_w_	0.96 ± 0.05	0.95 ± 0.03	0.96 ± 0.00	0.96 ± 0.00
Hardness [N]	30.08 ± 2.17 ^a^	31.03 ± 1.99 ^a^	35.89 ± 3.31 ^b^	38.94 ± 2.41 ^b^

Values followed by different small letters (^a^,^b^,^c^) in the same row are significantly different (*p* < 0.05). Values are means of three replicates ± standard deviation.

**Table 6:** Energy value and nutrient content in 100 g of designed breads.

Nutritional Value	C	CR5	CR10	CR15
Energy value (kJ/kcal)	877/211	890/213	904/216	916/219
Fat (g)	0.7	1.1	1.6	2.0
Carbohydrates (g)	39.8	38.1	36.5	34.8
Fibre (g)	5.8	5.7	5.7	5.6
Protein (g)	8.2	9.7	11.2	12.6
% energy from protein	15.7	18.2	20.7	23.0

**Table 7:** Microbiological analysis of tested breads after production and after 2 and 7 days of storage.

Samples	Days	TVC [log CFU g^−1^]	Y&M [log CFU g^−1^]
C	0	3.71 ± 0.28 ^a^	nd
	2	7.10 ± 0.01 ^b^	3.67 ± 0.02 ^a^
	7	8.39 ± 0.31 ^c^	4.33 ± 0.07 ^b^
CR5	0	4.30 ± 0.06 ^d^	nd
	2	6.49 ± 0.39 ^e^	nd
	7	7.37 ± 0.34 ^b^	4.34 ± 0.08 ^b^
CR10	0	4.52 ± 0.16 ^d^	nd
	2	7.19 ± 0.20 ^b^	3.43 ± 0.02 ^a^
	7	8.53 ± 0.20 ^c^	4.40 ± 0.11 ^b^
CR15	0	4.32 ± 0.12 ^d^	nd
	2	7.17 ± 0.19 ^b^	nd
	7	9.44 ± 0.29 ^f^	4.29 ± 0.08 ^b^

Values followed by different small letters (^a–f^) in the same column are significantly different (*p* < 0.05). Values are means of three replicates ± standard deviation. TVC—Total viable count; Y&M—yeast and moulds; nd—not detected.

**Table 8:** Recipes [%] for bread made with whole wheat flour (C), and whole wheat flour with the additions of 5% (CR5), 10% (CR10) and 15% (CR15) cricket powder.

Samples	Wholemeal Wheat Flour	Cricket Powder	Dried Yeasts	Sugar	Salt	Water
C	58.7	0.0	0.8	0.7	0.7	39.2
CR5	55.8	2.9	0.8	0.7	0.7	39.2
CR10	52.9	5.9	0.8	0.7	0.7	39.2
CR15	49.9	8.8	0.8	0.7	0.7	39.2

## Data Availability

Data are contained within the article.

## References

[1] Van Huis A., Van Itterbeeck J., Klunder H., Mertens E., Halloran A., Muir G., Vantomme P. (2013). Edible Insects: Future Prospects for Food and Feed Security.

[2] Rumpold B.A., Schlüter O.K. (2013). Nutritional composition and safety aspects of edible insects. Mol. Nutr. Food Res..

[3] Zielińska E., Baraniak B., Karaś M., Rybczyńska K., Jakubczyk A. (2015). Selected species of edible insects as a source of nutrient composition. Food Res. Int..

[4] Raheem D., Carrascosa C., Oluwole O.B., Nieuwland M., Saraiva A., Millán R., Raposo A. (2019). Traditional consumption of and rearing edible insects in Africa, Asia and Europe. Crit. Rev. Food Sci. Nutr..

[5] Sogari G., Bogueva D., Marinova D. (2019). Australian consumers’ response to insects as food. Agriculture.

[6] Jongema Y. (2017). Worldwide List of Recorded Edible Insects.

[7] Orkusz A. (2021). Edible Insects versus Meat—Nutritional Comparison: Knowledge of Their Composition Is the Key to Good Health. Nutrients.

[8] Abro Z., Kassie M., Tanga C., Beesigamukama D., Diiro G. (2020). Socio-economic and environmental implications of replacing conventional poultry feed with insect-based feed in Kenya. J. Clean. Prod..

[9] Finke M.D., Oonincx D.G.A.B., Van Huis A., Tomberlin J.K. (2017). Nutrient content of insects. Insects as Food and Feed: From Production to Consumption.

[10] Oonincx D.G.A.B., Dierenfeld E.S. (2012). An investigation into the chemical composition of alternative invertebrate prey. Zoo Biol..

[11] Oonincx D.G.A.B., Van der Poel A.F. (2011). Effects of diet on the chemical composition of migratory locusts (*Locusta migratoria*). Zoo Biol..

[12] Finke M.D. (2013). Complete nutrient content of four species of feeder insects. Zoo Biol..

[13] Cruz-López S.O., Escalona-Buendía H.B., Román-Guerrero A., Domínguez-Soberanes J., Alvarez-Cisneros Y.M. (2022). Charactezation of cooked meat models using grasshopper (*Sphenarium purpurascens*) soluble protein extracted by alkalisation and ultrasound as meat-extender. Food Sci. Anim. Resour..

[14] van Huis A., Rumpold B., Maya C., Roos N. (2021). Nutritional qualities and enhancement of edible insects. Annu. Rev. Nutr..

[15] Megido R.C., Gierts C., Blecker C., Brostaux Y., Haubruge É., Alabi T., Francis F. (2016). Consumer acceptance of insect-based alternative meat products in Western countries. Food Qual. Prefer..

[16] Skotnicka M., Karwowska K., Kłobukowski F., Borkowska A., Pieszko M. (2021). Possibilities of the development of edible insect-based foods in Europe. Foods.

[17] Regulation (EU) 2021/882. https://eur-lex.europa.eu/eli/reg_impl/2021/882/oj.

[18] Regulation (EU) 2021/1975. https://eur-lex.europa.eu/eli/reg_impl/2021/1975/oj.

[19] Regulation (EU) 2022/169. https://eur-lex.europa.eu/eli/reg_impl/2022/169/oj.

[20] Regulation (EU) 2022/188. https://eur-lex.europa.eu/eli/reg_impl/2022/188/oj.

[21] Regulation (EU) 2023/58. https://eur-lex.europa.eu/eli/reg_impl/2023/58/oj.

[22] Fernandez-Cassi X., Supeanu A., Vaga M., Jansson A., Boqvist S., Vagsholm I. (2019). The house cricket (*Acheta domesticus*) as a novel food: A risk profile. J. Insects Food Feed..

[23] Kulma M., Kouřimská L., Plachý V., Božik M., Adámková A., Vrabec V. (2019). Effect of sex on the nutritional value of house cricket, *Acheta domestica* L.. Food Chem..

[24] Kouřimská L., Kotrbová V., Kulma M., Adámková A., Mlček J., Sabolová M., Homolková D. (2020). Attitude of assessors in the Czech Republic to the consumption of house cricket *Acheta domestica* L.—A Prelim. Study. Czech J. Food Sci..

[25] Tan H.S.G., Fischer A.R., Tinchan P., Stieger M., Steenbekkers L.P.A., van Trijp H.C. (2015). Insects as food: Exploring cultural exposure and individual experience as determinants of acceptance. Food Qual. Prefer..

[26] Hartmann C., Siegrist M. (2017). Insects as food: Perception and acceptance. Findings from current research. Ernahr. Umsch..

[27] de Oliveira L.M., da Silva Lucas A.J., Cadaval C.L., Mellado M.S. (2017). Bread enriched with flour from cinereous cockroach (*Nauphoeta cinerea*). Innov. Food Sci. Emerg. Technol..

[28] Lucas-González R., Fernández-López J., Pérez-Álvarez J.A., Viuda-Martos M. (2019). Effect of drying processes in the chemical, physico-chemical, techno-functional and antioxidant properties of flours obtained from house cricket (*Acheta domesticus*). Eur. Food Res. Technol..

[29] Mafu A., Ketnawa S., Phongthai S., Schönlechner R., Rawdkuen S. (2022). Whole Wheat Bread Enriched with Cricket Powder as an Alternative Protein. Foods.

[30] Kowalski S., Mikulec A., Mickowska B., Skotnicka M., Mazurek A. (2022). Wheat bread supplementation with various edible insect flours. Influence of chemical composition on nutritional and technological aspects. LWT.

[31] Tiwari A., Jha S.N. (2017). Extrusion cooking technology: Principal mechanism and effect on direct expanded snacks—An overview. Int. J. Food Stud..

[32] Dinh T.T.N., To K.V., Schilling M.W. (2021). Fatty Acid Composition of Meat Animals as Flavor Precursors. Meat Muscle Biol..

[33] Gantner M., Król K., Piotrowska A., Sionek B., Sadowska A., Kulik K., Wiącek M. (2022). Adding Mealworm (*Tenebrio molitor* L.) Powder to Wheat Bread: Effects on Physicochemical, Sensory and Microbiological Qualities of the End-Product. Molecules.

[34] Khuenpet K., Pakasap C., Vatthanakul S., Kitthawee S. (2020). Effect of larval-stage mealworm (*Tenebrio molitor*) powder on qualities of bread. Int. J. Agric. Technol..

[35] Bartkiene E., Starkute V., Katuskevicius K., Laukyte N., Fomkinas M., Vysniauskas E., Kasciukaityte P., Radvilavicius E., Rokaite S., Medonas D. (2022). The contribution of edible cricket flour to quality parameters and sensory characteristics of wheat bread. Food Sci. Nutr..

[36] González C.M., Garzón R., Rosell C.M. (2019). Insects as ingredients for bakery goods. A comparison study of *H. illucens*, *A. domestica* and *T. molitor* flours. Food Sci. Emerg. Technol..

[37] Bellary A.N., Indiramma A.R., Prakash M., Baskaran R., Rastogi N.K. (2016). Anthocyanin infused watermelon rind and its stability during storage. Innov. Food Sci. Emerg. Technol..

[38] Villarino C.B., Jayaena V., Coorey R., Chakrabarti-Bell S., Johnson S.K. (2016). Nutritional, Health, and Technological Functionality of Lupin Flour Addition to Bread and Other Baked Products: Benefits and Challenges. Crit. Rev. Food Sci. Nutr..

[39] García-Segovia P., Igual M., Martínez-Monzó J. (2020). Physicochemical Properties and Consumer Acceptance of Bread Enriched with Alternative Proteins. Foods.

[40] Sui X., Zhang Y., Zhou W. (2016). Bread fortified with anthocyanin-rich extract from black rice as nutraceutical sources: Its quality attributes and in vitro digestibility. Food Chem..

[41] Ruszkowska M., Tańska M., Kowalczewski P.Ł. (2022). Extruded Corn Snacks with Cricket Powder: Impact on Physical Parameters and Consumer Acceptance. Sustainability.

[42] Regulation (EU) No. 1924/2006 of the European Parliament and of the Council of 20 December 2011 on Nutrition and Health Claims Made on Foods. https://eur-lex.europa.eu/LexUriServ/LexUriServ.do?uri=OJ:L:2006:404:0009:0025:En:PDF.

[43] Wieczorek M.N., Kowalczewski P.Ł., Drabińska N., Różańska M.B., Jeleń H.H. (2022). Effect of Cricket Powder Incorporation on the Profile of Volatile Organic Compounds, Free Amino Acids and Sensory Properties of Gluten-Free Bread. Pol. J. Food Nutr. Sci..

[44] Nowakowski A.C., Miller A.C., Miller M.E., Xiao H., Wu X. (2022). Potential health benefits of edible insects. Crit. Rev. Food Sci. Nutr..

[45] Aguilar-Toalá J.E., Cruz-Monterrosa R.G., Liceaga A.M. (2022). Beyond Human Nutrition of Edible Insects: Health Benefits and Safety Aspects. Insects.

[46] Kipkoech C. (2023). Beyond Proteins—Edible Insects as a Source of Dietary Fiber. Polysaccharides.

[47] Borges M.M., da Costa D.V., Trombete F.M., Câmara A.K.F.I. (2022). Edible insects as a sustainable alternative to food products: An insight into quality aspects of reformulated bakery and meat products. Curr. Opin. Food Sci..

[48] Roncolini A., Milanović V., Cardinali F., Osimani A., Garofalo C., Sabbatini R., Clementi F., Pasquini M., Mozzon M., Foligni R. (2019). Protein fortification with mealworm (*Tenebrio molitor* L.) powder: Effect on textural, microbiological, nutritional and sensory features of bread. PLoS ONE.

[49] Osimani A., Milanović V., Cardinali F., Roncolini A., Garofalo C., Clementi F., Pasquini M., Mozzon M., Foligni R., Raffaelli N. (2018). Bread enriched with cricket powder (*Acheta domesticus*): A technological, microbiological and nutritional evaluation. Innov. Food Sci. Emerg. Technol..

[50] Malomo O., Ogunmoyela O.A.B., Oluwajoba S.O., Dudu O.E. (2012). Microbiological and nutritional quality of warankashi enriched bread. J. Microbiol. Biotechnol. Food Sci..

[51] Garcia M.W., Bregão A.S., Parussolo G., Bernardi A.O., Stefanello A., Copetti M.V. (2019). Incidence of spoilage fungi in the air of bakeries with different hygienic status. Int. J. Food Microbiol..

[52] Deschuyffeleer N., Audenaert K., Samapundo S., Ameye S., Eeckhout M., Devlieghere F. (2011). Identification and characterization of yeasts causing chalk mould defects on par-baked bread. Food Microbiol..

[53] Belz M.C., Mairinger R., Zannini E., Ryan L.A., Cashman K.D., Arendt E.K. (2012). The effect of sourdough and calcium propionate on the microbial shelf-life of salt reduced bread. Appl. Microbiol. Biotechnol..

[54] Garofalo C., Zannini E., Aquilanti L., Silvestri G., Fierro O., Picariello G., Clementi F. (2012). Selection of sourdough lactobacilli with antifungal activity for use as biopreservatives in bakery products. J. Agric. Food Chem..

[55] Dagnas S., Membré J.-M. (2013). Predicting and Preventing Mold Spoilage of Food Products. J. Food Prot..

[56] Giannone V., Pitino I., Pecorino B., Todaro A., Spina A., Lauro M.R., Tomaselli F., Restuccia C. (2016). Effects of innovative and conventional sanitizing treatments on the reduction of Saccharomycopsis fibuligera defects on industrial durum wheat bread. Int. J. Food Microbiol..

[57] Hernández A., Pérez-Nevado F., Ruiz-Moyano S., Serradilla M.J., Villalobos M.C., Martis A., Córdoba M.G. (2018). Spoilage yeasts: What are the sources of contamination of foods and beverages?. Int. J. Food Microbiol..

[58] Quattrini M., Liang N., Fortin M.G., Xiang S., Curtis J.M., Gänzle M. (2019). Exploiting synergies of sourdough and antifungal organic acids to delay fungal spoilage of bread. Int. J. Food Microbiol..

[59] El Houssni I., Khedid K., Zahidi A., Hassikou R. (2023). The inhibitory effects of lactic acid bacteria isolated from sourdough on the mycotoxigenic fungi growth and mycotoxins from wheat bread. Biocatal. Agric. Biotechnol..

[60] Dymchenko A., Geršl M., Gregor T. (2023). Trends in bread waste utilization. Trends Food Sci. Technol..

[61] Garcia M.W., Bernardi A.O., Copetti M.V. (2019). The fungal problem in bread production: Insights of causes, consequences, and control methods. Curr. Opin. Food Sci..

[62] Dos Santos J.L.P., Bernardi A.O., Pozza Morassi L.L., Silva B.S., Copetti M.V., Sant’Ana A.S. (2016). Incidence, populations and diversity of fungi from raw materials, final products and air of processing environment of multigrain whole meal bread. Food Res. Int..

[63] Caro I., Portales S., Gómez M. (2023). Microbial characterization of discarded breads. LWT.

[64] Kunachowicz H., Przygoda B., Nadolna I., Iwanow K. (2023). Tabele Składu i Wartości Odżywczej Żywności.

[65] (2013). Microbiology of the Food Chain. Horizontal Method for the Enumeration of Microorganisms. Part 1: Colony Count at 30 °C by the Pour Plate Technique.

[66] (2008). Microbiology of Food and Animal Feeding Stuffs—Horizontal Method for the Enumeration of Yeasts and Moulds—Part 2: Colony Count Technique in Products with Water Activity Less than or Equal to 0.95.

[67] (2003). Sensory Analysis. Guidelines for the Use of Quantitative Response Scales.

[68] (2012). Sensory Analysis. General Guidelines for the Selection, Training and Monitoring of Selected Assessors and Expert Sensory Assessors.

[69] (2010). Sensory Analysis. General Guidance for the Design of Test Rooms.

